# Associations Between Antithrombosis and Ventilator-Associated Events, ICU Stays, and Mortality Among Mechanically Ventilated Patients: A Registry-Based Cohort Study

**DOI:** 10.3389/fphar.2022.891178

**Published:** 2022-07-18

**Authors:** Mingqi Wang, Wen Wang, Xue Jia, Qiao He, Shichao Zhu, Yan Kang, Rui Zhang, Yan Ren, Ling Li, Kang Zou, Zhiyong Zong, Xin Sun

**Affiliations:** ^1^ Chinese Evidence-based Medicine Center and Cochrane China Center, West China Hospital, Sichuan University, Chengdu, China; ^2^ NMPA Key Laboratory for Real World Data Research and Evaluation in Hainan, West China Hospital, Sichuan University, Chengdu, China; ^3^ Sichuan Center of Technology Innovation for Real World Data, Chengdu, China; ^4^ Department of Postgraduate, West China Hospital of Sichuan University, Chengdu, China; ^5^ Department of Infection Control, West China Hospital of Sichuan University, Chengdu, China; ^6^ Intensive Care Unit, West China Hospital of Sichuan University, Chengdu, China; ^7^ Information Center, West China Hospital, Sichuan University, Chengdu, China; ^8^ Center of Infection Diseases, West China Hospital of Sichuan University, Chengdu, China

**Keywords:** antithrombosis prophylaxis, ventilator-associated events, ventilator-associated pneumonia, ICU mortality, patients receiving mechanical ventilation

## Abstract

**Background:** The effect of thromboembolism prophylaxis on clinical outcomes, such as ventilator-associated events (VAEs), ICU stays, and mortality, remains controversial. This study was conducted to evaluate the effect of pharmacological thromboprophylaxis on VAEs, ICU stays, and ICU mortality among patients receiving mechanical ventilation (MV).

**Materials and Methods:** A retrospective cohort study was conducted based on a well-established registry of healthcare-associated infections at ICUs in the West China Hospital system. Patients who consistently received MV for at least 4 days from 1 April 2015 to 31 December 2018 were included. Hazard ratios (HRs) were compared for three tiers of VAEs, ICU stays, and ICU mortality among patients receiving pharmacological thromboprophylaxis versus those without using the time-dependent Cox model. For the analyses of ICU stays and ICU mortality, we also used Fine-Gray models to disentangle the competing risks and outcomes of interest.

**Results:** Overall, 6,140 patients were included. Of these, 3,805 received at least one prescription of antithrombosis agents. Treatments with antithrombosis agents were associated with lower risk of VAEs (HR: 0.87, 95% CI: 0.77, 0.98) and ICU mortality (HR: 0.72, 95% CI: 0.61, 0.86) than those without. Anticoagulants but not antiplatelet agents were associated with decreased risk of VAEs (HR: 0.86, 95% CI: 0.75, 0.98), ICU mortality (HR: 0.62, 95% CI: 0.51, 0.76), and less time to ICU discharge (HR: 1.15, 95% CI: 1.04, 1.28). Antithrombosis may be associated with decreased risk of VAEs in patients with D-dimer >5 mg/LFEU (HR: 0.84, 95%CI: 0.72, 0.98).

**Conclusions:** Pharmacological thromboprophylaxis was associated with lower risk of VAEs and ICU mortality. Similar effects were observed between unfractionated heparins versus low-molecular-weight heparins.

## Background

Ventilator-associated event (VAE) and ventilator-associated pneumonia (VAP) are important causes of morbidity and mortality in the ICU (Bouadma et al., 2015; Magill et al., 2016; He et al., 2021; Zhu et al., 2021). To improve clinical outcomes of patients receiving mechanical ventilation, ventilator bundles have been implemented by hospitals worldwide. Although ventilator bundles have been implemented by hospitals worldwide to prevent adverse outcomes (Marra et al., 2009; Croce et al., 2013), the components often vary remarkably, which may be associated with adverse outcomes.

As a core constituent of ventilator bundles, thromboembolism prophylaxis has been advocated by the Institute for Healthcare Improvement and widely used by most hospitals (Klompas, 2010; Klompas et al., 2016). Owing to immobilization, venous stasis, and vascular injury, venous thromboembolism (VTE) is common among intensive care unit (ICU) patients (Attia et al., 2001; Boddi and Peris, 2017; Ejaz et al., 2018). Patient prognoses are often poor with VTE, especially deep vein thrombosis (DVT) and pulmonary embolism (PE) (Jain and Schmidt, 1997; Laporte and Mismetti, 2010; Ejaz et al., 2018). Thromboembolism prophylaxis has been proven to reduce risk of VTE (Jain and Schmidt, 1997; Boonyawat and Crowther, 2015; Boddi and Peris, 2017). In particular, pharmacological thromboprophylaxis has been recommended for ICU patients to lower risk of VTE (Boddi and Peris, 2017).

However, the effects of thromboembolism prophylaxis on other adverse outcomes such as VAE and mortality have not been fully understood (Al Yami et al., 2017; Bajaj et al., 2019). The benefit of reducing VTE should be reappraised given the increased risk of adverse outcomes, such as major bleeding. Especially for patients with MV more than 48 h, the risk of major bleeding significantly increased (Goodwin and Hoffman, 2011), which may further result in poor prognosis including infection and mortality. Few studies addressed these important outcomes, and these studies draw inconsistent conclusions (Barrera et al., 2010; Klemen et al., 2020). An observational study involved 630 patients with at least 2 days of MV, of which 210 patients developed ventilator-associated pneumonia (VAP). The findings of this study have showed that thromboprophylaxis was significantly associated with lower risk of VAP and death (Croce et al., 2013). Several other studies reported no association between pharmacological thromboprophylaxis on VAP and VAE (Berenholtz et al., 2011; Lewis et al., 2014; Klompas et al., 2016). Most of these studies had relatively small sample size and only a few events occurred, which may have led to a limited inference. For instance, a case–control study only included 110 ventilator-associated condition (VAC) and 38 infection-related ventilator-associated complication (IVAC) to investigate the risk factors for VACs and IVACs, respectively (Lewis et al., 2014). In addition, most studies have exclusively considered implementing aggregate bundle components together, rather than considering day-to-day implementation of bundles (Croce et al., 2013; Harris et al., 2018). Indeed, the implementation of bundle components varied on a day-to-day basis (Klompas et al., 2016).

Therefore, we conducted a cohort study with a large sample size and handled with time-dependent variates to evaluate the effect of prophylactic antithrombosis agents on clinical outcomes.

## Materials and Methods

This cohort study was reported according to the Reporting of studies Conducted using Observational Routinely collected health Data (Benchimol et al., 2015). This study was approved by the Ethics Committee of West China Hospital (WCH) in 2018 (WCH2018-409).

### Data Source

This study was conducted based on a registry of healthcare-associated infections (HAI) at ICUs in WCH system. The WCH is a major healthcare system in west China, which comprises three independent healthcare organizations (Main Campus, Wenjiang Campus, and Shangjin Campus). A total of 5.73 million outpatients and 279,000 inpatients visited to WCH in 2019. As a national critical care center in west China, the WCH has six ICUs (general ICU, surgical ICU, neurological ICU, respiratory ICU, thoracic surgery ICU, and pediatric ICU) with more than 8,000 inpatient ICU admissions annually.

The ICU-HAI registry contained three databases—ICU system, electronic medical record (EMR), and ICU-HAI system. The ICU-HAI system is an active surveillance system, which is a unique system undertaking routine VAE surveillance in China. Information regarding catheterization, hospital-acquired infection, prevention, and control are collected by a team of experienced infection control practitioners every day. Every year, there were more than 5,000 patients undertake VAE surveillance. Of these, 2,000 patients received MV for at least four consecutive day and 500 patients were judged as VAE cases annually. By integrating these three databases with unique and encoded identifiers, we developed the ICU-HAI registry (Li et al., 2018; He et al., 2021).

Patients who were admitted to the six ICUs in WCH since 1 April 2015 were included into the registry. The registry included 28,848 patients until 31 December 2018 and contained 110 GB of data with 245, 311, 294 original records (Wang et al., 2019). Quality assessment showed that the accuracy of data extraction and linkage was 100%, and the completeness of important laboratory tests such as routine blood tests, serum glucose, and serum creatinine was >98% (Wang et al., 2019).

### Study Population

Patients who consistently received MV for at least 4 days from 1 April 2015 to 31 December 2018 were included in the study. The exclusion criteria were as follows: age <18 years, patients admitted to PICU, without sufficient information regarding age, sex, and diagnosis at discharge, and with a diagnosis related to venous thrombosis at ICU admission. The clinical characteristics differed among non-VAE cases with and without consecutive stable or improved respiratory status. Therefore, we also excluded patients with consecutive unstable or increasing daily minimum positive end-expiratory pressure (PEEP) or fraction of inspired oxygen (FiO2) during MV treatment. To minimize indication bias, we additionally excluded patients with extremely long ICU stays (>90 days) and patients with more than one episode of MV treatment for the analysis of ICU mortality and ICU stays.

### Antithrombosis Agent Exposure

We defined antithrombosis agent exposure as a prescription for antiplatelet or anticoagulant agents. We calculated antithrombosis agent exposure as a time-dependent variable. Daily exposure to antiplatelet agents or anticoagulant agents were coded as prescribed or not prescribed from initiation of MV to VAE occurrence or extubation and from ICU admission to ICU discharge, respectively. We additionally evaluated the type of antithrombosis agents (antiplatelet and anticoagulant agent) and type of anticoagulant agents [unfractionated heparin (UFH) and low molecular weight heparin (LMWH)] used.

### Clinical Outcomes

The clinical outcomes of interest included VAEs, ICU mortality, and ICU stays. We measured clinical outcomes as time-to-event variables. We defined VAEs as at least 2 calendar days of increased daily minimum FiO_2_ (≥0.20) or PEEP (≥3 cm H_2_O) greater than after at least 2 calendar days of stable or decreasing daily minimum FiO_2_ or PEEP according to the criteria proposed by CDC’s National Healthcare Safety Network (CDC-NHSN) (Klompas, 2013). Once the value reached the threshold, an alarm would be triggered. Then, infection control practitioners would judge and classify the suspected VAE cases as VACs, IVACs, and possible ventilator-associated pneumonia (PVAP). The accuracy of PVAP has been validated in a previous study, and the proportion was 96.2% (Wang et al., 2019). Patients died within one calendar day after ICU discharge were defined as ICU mortality.

### Statistical Analysis

We assessed hazards ratio (HR) with confidence interval (CI) for antithrombosis agents and risk of VAEs by using time-dependent Cox model. This model is used to analyze studies with complex time-varying variates, and data were converted as counting process form to deal with time-varying variates. We assessed the impact of antithrombosis agents on ICU mortality and ICU stays using Fine‐Gray competing risks model to measure the competing risks for ICU discharge alive versus ICU mortality. The reason for ICU discharge was depended on patient’s health condition: clinical improvement or death. Through generating separate hazard ratios for each competing events, Fine‐Gray competing risks model can disentangle effects of competing risks and outcomes of interest (Fine and Gray, 1999; Berger et al., 2018).

All analyses were adjusted for fixed and time-varying covariates. We defined ICU type, demographic characteristics, comorbidities or acute condition (diabetes, hypertension, heart failure, kidney failure, liver failure, ischemic heart disease, cerebrovascular diseases, chronic obstructive pulmonary disease, pulmonary vascular diseases, malignant tumor, trauma, acute respiratory distress syndrome, shock, gastrointestinal bleeding, pneumonia, and intra-abdominal infection), acute physiology and chronic health evaluation (APACHE) Ⅱ score, surgery, fiberoptic bronchoscopy examination, tracheotomy, and laboratory test at admission (D-dimer, prothrombin time, platelet count, antithrombin III, activated partial thromboplastin time) as fixed covariates. Daily medication exposure and processes of care (sedative, acid inhibitors, blood transfusion, mandatory ventilation, and head-of-bed elevation, gastrointestinal decompression, and rehabilitation exercise) and medications (sedatives, opioids, neuromuscular blockers, immunosuppressive agent, neuroleptic agents, antibiotics, expectorants, vasopressors, intestinal probiotics, and neuroleptic agents) were defined as time-varying variables. Time-varying variables were measured as daily exposure from initiation of MV to the event of interested for the model of VAEs and each day from ICU admission to ICU discharge for the model of ICU discharge and ICU mortality. For the analysis of ICU discharge and ICU mortality, we additionally adjusted VAE, days from ICU admission to initiation of MV, and the duration of mechanical ventilation. We also measured mandatory ventilation and prone position ventilation as fixed-time variables.

To evaluate the effect of different types of antithrombosis agents on clinical outcomes, we further calculated HRs for antiplatelet agents vs. regimens without antithrombosis agents, anticoagulant agents vs. regimens without antithrombosis agents, antiplatelet agents vs. anticoagulant agents, and UFH vs. LMWH. We also calculated HRs regarding VAEs, ICU mortality, and ICU stays for patients with D-dimer ≤5 mg/L and >5 mg/L, respectively. The HRs for VAEs were assessed using time-dependent Cox model. We also used Fine‐Gray competing risks model to estimate HRs for ICU mortality and length of ICU stay. Missing data of APACHE II, prothrombin time, D-dimer, platelet count, and lipid load were processed using multilevel multiple imputation. Through pooling results from different imputed datasets, multiple imputations can help reduce bias and obtain more precise results compared with complete-case analysis.

Significance is 0.05 for all analysis, and all data were analyzed using R version 4.0.3, the packages used mainly included “survival,” “miceadds,” “mice.”

### Sensitivity Analyses and Additional Analyses

We conducted the following sensitivity analyses to examine the robustness of effect estimates: 1) alternative statistical models: without adjusting prothrombin time, platelet count, antithrombin III, activated partial thromboplastin time; 2) alternative approach for missing data: missing data without imputation; and 3) alternative definition of comparison: regimens without anticoagulant agents and regimens without antiplatelet agents.

To further evaluate the effect of antithrombosis agents on VTE, we conducted an additional analysis. We excluded patients who developed VTE within 3 days after ICU admission for latency purpose and to minimize reverse causality. We assessed HRs for antithrombosis agents and risk of VTE using time-dependent Cox model.

## Results

**FIGURE 1:**
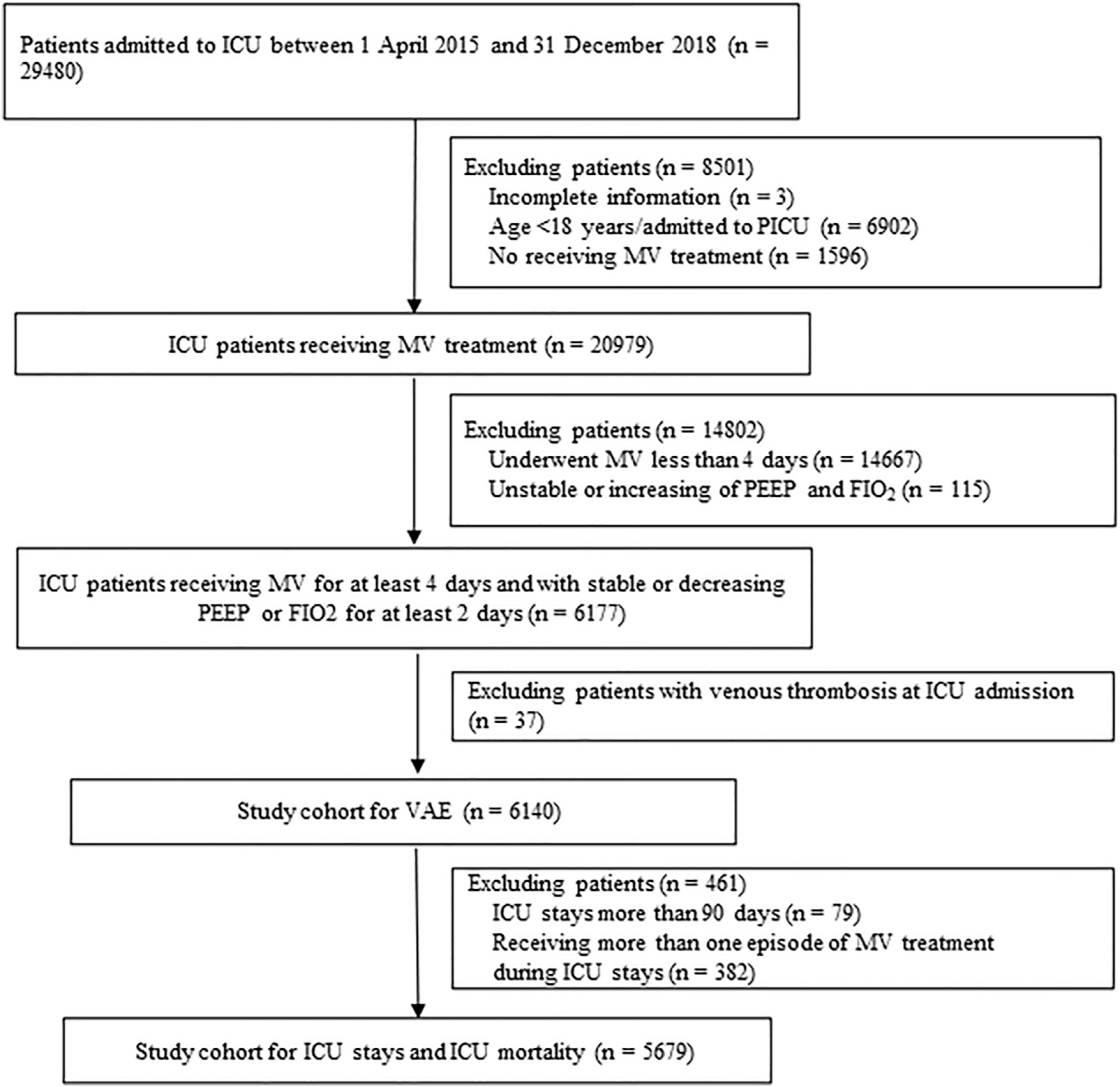
Study flow chart. ICU: intensive care units; MV: Mechanical ventilation; PEEP: Positive end-expiratory pressure; FIO_2_: Fraction of inspired oxygen; VAE: Ventilator-associated events

Our study included 6,140 patients consistently received MV for at least 4 days. Of these, 5,679 patients with ICU stay less than 90 days and one episode of MV treatment was additionally included into the cohort for ICU stays and ICU mortality analysis (Figure 1). Of included 6,140 patients, 3,805 received at least one prescription of antithrombosis agents and 2,335 did not receive any prescription of antithrombosis agents.

Patients’ characteristics are showed in Table1. The median age of all included patients was 58 years, and female patients accounted for 36.8% of subjects among included patients. The distribution of patients with and without antithrombosis agents treatment in different ICU wards showed a significant difference (*p* < 0.001). For instance, 29.2 and 38.7% patients with and without antithrombosis agents treatment, respectively, were admitted to the general ICU. The median APACHEII score was lower among patients treated with antithrombosis agents than those without [19 (15, 24) vs. 21 (16, 26), *p* < 0.001]. Among all included patients, the most common comorbidity was hypertension (21.5%) and the most common acute condition was pneumonia (10.2%). Compared to patients without antithrombosis agents treatment, the proportions of patients with heart failure (12.7 vs. 2.1%), ischemic heart disease (2.1 vs. 1.2%), pulmonary vascular disease (9.2 vs. 5.7%), and intra-abdominal infection (5.9 vs. 2.8%) were higher among patients receiving antithrombosis agents (*p* < 0.05). Among patients treated with antithrombosis agents, the activated partial thromboplastin time [33.6 s (28.9, 41.9) vs. 34.7 s (29.1, 44.5)] was higher, and antithrombin III [66.4 s (50.5, 81.3) vs. 62.0 s (46.6, 76.4)] was lower than patients who did not receive antithrombosis agents (*p* < 0.05).

**TABLE 1:** Baseline characteristics of patients.

	Overall (n = 6,140)	Antithrombosis agents users (n = 3,805)	Nonantithrombosis agents users (n = 2,335)	*p* value
Age (median (IQR])	58 [46, 70]	58 [47, 69]	58 [45, 70]	0.222
18–44	1,368 (22.3)	795 (20.9)	573 (24.5)	
45–59	1821 (29.7)	1,176 (30.9)	645 (27.6)	
60–74	1942 (31.6)	1,225 (32.2)	717 (30.7)	
≥75	1,009 (16.4)	609 (16.0)	400 (17.1)	
Female (n, %)	2,259 (36.8)	1,397 (36.7)	862 (36.9)	0.895
ICU Ward (%)				<0.001
GICU	2014 (32.8)	1,111 (29.2)	903 (38.7)	
NICU	1,165 (19.0)	633 (16.6)	532 (22.8)	
RICU	1,022 (16.6)	527 (13.9)	495 (21.2)	
SICU	1,201 (19.6)	807 (21.2)	394 (16.9)	
TICU	738 (12.0)	727 (19.1)	11 (0.5)	
APACHEII score (median (IQR])	20 [15, 25]	19 [15, 24]	21 [16, 26]	<0.001
Comorbidities/Acute conditions (%)				
Diabetes	517 (8.4)	307 (8.1)	210 (9.0)	0.222
Hypertension	1,322 (21.5)	843 (22.2)	479 (20.5)	0.137
Heart failure	531 (8.6)	482 (12.7)	49 (2.1)	<0.001
Kidney failure	414 (6.7)	223 (5.9)	191 (8.2)	0.001
Liver failure	134 (2.2)	61 (1.6)	73 (3.1)	<0.001
Ischemic heart disease	110 (1.8)	81 (2.1)	29 (1.2)	0.015
Brain hemorrhage	1,397 (22.8)	821 (21.6)	576 (24.7)	0.006
Ischemic stroke	462 (7.5)	318 (8.4)	144 (6.2)	0.002
Other hemorrhagic diseases	585 (9.5)	308 (8.4)	277 (11.9)	<0.001
COPD	677 (11.0)	387 (10.2)	290 (12.4)	0.007
Pulmonary vascular disease	483 (7.9)	350 (9.2)	133 (5.7)	<0.001
Malignant Tumor	465 (7.6)	276 (7.3)	189 (8.1)	0.246
Trauma	747 (12.2)	465 (12.2)	282 (12.1)	0.899
ARDS	73 (1.2)	44 (1.2)	29 (1.2)	0.858
Shock	344 (5.6)	230 (6.0)	114 (4.9)	0.062
Gastrointestinal bleeding	99 (1.6)	57 (1.5)	42 (1.8)	0.422
Sepsis	405 (6.6)	248 (6.5)	157 (6.7)	0.793
Pneumonia	625 (10.2)	359 (9.4)	266 (11.4)	0.016
Intra-abdominal infection	288 (4.7)	223 (5.9)	65 (2.8)	<0.001
Cardiac surgery (%)	487 (9.9)	371 (11.4)	116 (7.1)	<0.01
Cranial surgery (%)	446 (9.1)	315 (9.6)	131 (8.0)	0.060
Laboratory tests at Admission (median (IQR])				
D-dimer (mg/L)	5.46 [2.61, 11.19]	5.29 [2.50, 11.19]	5.64 [2.79, 11.20]	0.334
APTT (s)	34.30 [29.00, 43.40]	34.7 [29.10, 44.50]	33.6 [28.90, 41.90]	<0.001
Antithrombin III (mg/dl)	63.50 [48.10, 78.50]	62.00 [46.60, 76.40]	66.40 [50.50, 81.30]	<0.001
Prothrombin time (s)	13.70 [12.40, 15.60]	13.70 [12.40, 15.60]	13.70 [12.40, 15.70]	0.711
Platelets count (*10^9^/L)	130 [85, 196]	130 [88, 193]	131 [80, 201]	0.261
Decreased platelets (%)	1,194 (19.4)	675 (17.9)	519 (22.5)	<0.001
Days before MV initiation (median (IQR])	1 [0, 5]	1 [0, 6]	0 [0, 4]	<0.001
VAE (n, %)	1723 (28.1)	970 (25.5)	753 (32.2)	<0.001
IVAC	498 (28.9)	292 (30.1)	206 (27.4)	0.013
VAC	1,057 (61.3)	569 (58.7)	488 (64.8)	
PVAP	168 (9.8)	109 (11.2)	59 (7.8)	
Length of ICU stay (median (IQR])	13 [8, 22]	13 [7, 23]	13 [8, 21]	0.001
ICU death (n, %)	810 (14.3)	452 (12.3)	358 (17.9)	<0.001

GICU: general intensive care unit; NICU: neurological intensive care unit; RICU: respiratory intensive care unit; SICU: surgery intensive care unit; TICU: thoracic intensive care unit; APACHE II: acute physiology and chronic health evaluation; COPD: chronic obstructive pulmonary disease; ARDS: acute respiratory distress syndrome; APTT: activated partial thromboplastin time; MV: mechanical ventilation; VAE: ventilator-associated events; IVAC:infection-related ventilator-associated complication; VAC: ventilator-associated condition; VAP: ventilator-associated pneumonia; ICU: intensive care units.

Among 6,140 included patients, 1,723 patients experienced at least one episode of VAEs. Of these, 498 were classified as IVACs and 168 as PVAPs. The median of ICU stays was 13 days [8, 22], and the crude ICU mortality was 14.3% among included patients.

### Use of Antithrombosis Agents

A total of 3,805 patients (62.0%) received at least one prescription of antithrombosis agent among all included patients. Of those, 691 patients (11.3%) were treated with antiplatelet agents and 3,517 (57.3%) were treated with anticoagulants. Furthermore, 2,577 (42.0%) patients received UFH treatment, and 1,396 (22.7%) received LMWH treatment (Table 2).

**TABLE 2:** Use of antithrombotic agents among study population.

	Overall (n = 6,140)
Antithrombotic agents (n, %)	3,805 (62.0)
Anticoagulant agents (n, %)	3,517 (57.3)
Anticoagulant agents only	3,114 (50.7)
Heparin (n, %)	3,497 (57.0)
UFH only	2,101 (34.2)
LMWH only	920 (15.0)
LMWH and UFH	476 (7.8)
Antiplatelet agents (n, %)	691 (11.3)
Antiplatelet agents only	288 (4.7)

UFH: unfractionated heparin; LWMH: low molecular weight heparin.

### Associations Between Antithrombosis Agents and VAEs

Adjusted HRs for antithrombosis agents and VAEs are summarized in Table 3. Regimens with antithrombotic (HR: 0.87, 95% CI: 0.77, 0.98) and anticoagulant (HR: 0.86, 95% CI: 0.75, 0.98) agents were associated with lower risk of VAEs than regimens without. However, no statistically significant decrease in VAEs was found for antiplatelet agents (HR: 0.92, 95% CI: 0.76, 1.11). There were no significant differences between anticoagulants and antiplatelets (HR: 0.92, 95% CI: 0.72, 1.17) and between LMWH and UFH (HR: 1.09, 95% CI: 0.85, 1.39), regarding hazards for VAEs.

**TABLE 3:** Association between antithrombotic agents and VAEs.

	Crude model for VAEs		Adjusted model for VAEs		Crude model for IVACs		Adjusted model for IVACs		Crude model for PVAPs		Adjusted model for PVAPs	
	HR (95%CI)	*p* value	HR (95%CI)	*p* value	HR (95%CI)	*p* value	HR (95%CI)	*p* value	HR (95%CI)	*p* value	HR (95%CI)	*p* value
Agent vs. no agent comparisons												
Antithrombotic agents vs. regimens without antithrombotic agents	0.80 (0.72, 0.88)	<0.001	0.87 (0.77, 0.98)	0.020	0.90 (0.76, 1.06)	0.205	0.90 (0.74, 1.08)	0.258	1.32 (0.97, 1.79)	0.082	1.00 (0.69, 1.46)	0.979
Anticoagulant agents vs. regimens without antithrombotic agents	0.78 (0.7, 0.88)	<0.001	0.86 (0.75, 0.98)	0.019	0.84 (0.70, 1.01)	0.068	0.84 (0.68, 1.04)	0.117	1.20 (0.85, 1.7)	0.311	0.92 (0.61, 1.41)	0.712
Antiplatelet agents vs. regimens without antithrombotic agents	0.84 (0.69, 1.03)	0.099	0.92 (0.76, 1.11)	0.376	1.03 (0.76, 1.41)	0.850	1.08 (0.82, 1.42)	0.597	1.23 (0.67, 2.27)	0.502	1.51 (0.91, 2.49)	0.110
Agent vs. agent comparisons												
Anticoagulant agents vs. antiplatelet agents	0.93 (0.75, 1.16)	0.530	0.92 (0.72, 1.17)	0.507	0.82 (0.58, 1.14)	0.237	0.76 (0.53, 1.10)	0.153	0.97 (0.51, 1.85)	0.930	0.64 (0.32, 1.3)	0.225
LMWH vs. UFH	0.82 (0.67, 0.99)	0.048	1.09 (0.85, 1.39)	0.486	0.60 (0.43, 0.82)	0.001	0.98 (0.66, 1.46)	0.932	0.36 (0.21, 0.63)	<0.001	0.81 (0.38, 1.72)	0.590

VAE: ventilator-associated events; IVAC: infection-related ventilator-associated complication; VAP: ventilator-associated pneumonia; UFH: unfractionated heparin; LWMH: low molecular weight heparin. Model adjusted for: age, sex, acute physiology and chronic health evaluation (APACHE) Ⅱ score, ICU type (general, surgical, neurological, respiratory, thoracic surgery and pediatric ICU), comorbidities or condition (diabetes, hypertension, heart failure, kidney failure, liver failure, ischemic heart disease, cerebrovascular diseases, chronic obstructive pulmonary disease, pulmonary vascular diseases, malignant tumor, trauma, acute respiratory distress syndrome, shock, gastrointestinal bleeding, pneumonia, and intra-abdominal infection), cardiac surgery, cranial surgery, fiberoptic bronchoscopy examination, tracheotomy, laboratory test at admission (D-dimer, prothrombin time, platelet count, antithrombin III, activated partial thromboplastin time), daily medication exposure and processes of care (sedative, acid inhibitors, blood transfusion, mandatory ventilation, and head-of-bed elevation, gastrointestinal decompression, rehabilitation exercise) and medications (sedatives, opioids, neuromuscular blockers, immunosuppressive agent, neuroleptic agents, antibiotics, expectorants, vasopressors, intestinal probiotics and neuroleptic agents).

Compared to regimens without antithrombosis agent, no statistically significant decrease in IVACs and PVAPs was found for antithrombotic, anticoagulation, and antiplatelet agent. There were no statistically differences regarding the comparisons of antiplatelet agents vs. regimens without antithrombosis agents, anticoagulation agents vs. antiplatelet agents, and LMWH vs. UFH (Table 3).

### Associations Between Antithrombosis Agents and the Length of ICU Stay and ICU Mortality

Adjusted HRs for antithrombosis agents and ICU mortality and length of stay are summarized in Table 4. Compared to regimens without antithrombosis agent, antithrombosis agent (HR: 0.72, 95% CI: 0.61, 0.86) and anticoagulant (HR: 0.62, 95%CI: 0.51, 0.76) agents were associated with lower risk of ICU mortality. Regimens with anticoagulant agents were associated an increased hazard for ICU discharge (HR: 1.15, 95%CI: 1.04, 1.27) than those without antithrombosis agent, suggesting that anticoagulant agents were associated with shorter time of ICU stays. Anticoagulant agents were associated with decreased risk of ICU mortality (HR:0.52, 95%CI: 0.37, 0.74) and less time to ICU discharge (HR:1.43, 95%CI: 1.18, 1.73) relative to antiplatelets agent. No statistically differences were found between LMWH and UFH regarding hazards for ICU mortality and ICU stays (Table 4).

**TABLE 4:** Association between antithrombotic agents and patient outcomes.

	Crude model for ICU mortality		Adjusted model for ICU mortality		Crude model for ICU stays		Adjusted model for ICU stays	
	HR (95% CI)	*p* value	HR (95% CI)	*p* value	HR (95% CI)	*p* value	HR (95% CI)	*p* value
Agent vs. no agent comparisons								
Antithrombotic agents vs. regimens without antithrombotic agents	0.69 (0.59, 0.8)	<0.001	0.72 (0.61, 0.86)	<0.001	1.69 (1.57, 1.81)	<0.001	1.07 (0.98, 1.18)	0.14
Anticoagulant agents vs. regimens without antithrombotic agents	0.45 (0.38, 0.54)	<0.001	0.62 (0.51, 0.76)	<0.001	1.82 (1.69, 1.97)	<0.001	1.15 (1.04, 1.27)	0.006
Antiplatelet agents vs. regimens without antithrombotic agents	0.94 (0.71, 1.24)	0.658	1.12 (0.86, 1.46)	0.406	0.89 (0.75, 1.05)	0.155	0.88 (0.76, 1.02)	0.079
Agent vs. agent comparisons								
Anticoagulant agents vs. antiplatelet agents	0.48 (0.35, 0.65)	<0.001	0.52 (0.37, 0.74)	<0.001	2.06 (1.74, 2.44)	<0.001	1.43 (1.18, 1.73)	<0.001
LMWH vs. UFH	1.03 (0.74, 1.44)	0.856	0.92 (0.62, 1.37)	0.683	0.51 (0.45, 0.57)	<0.001	1.02 (0.84, 1.23)	0.864

UFH: unfractionated heparin; LWMH: low molecular weight heparin. Model adjusted for: age, sex, acute physiology and chronic health evaluation (APACHE) Ⅱ score, ICU type (general, surgical, neurological, respiratory, thoracic surgery and pediatric ICU), comorbidities or condition (diabetes, hypertension, heart failure, kidney failure, liver failure, ischemic heart disease, cerebrovascular diseases, chronic obstructive pulmonary disease, pulmonary vascular diseases, malignant tumor, trauma, acute respiratory distress syndrome, shock, gastrointestinal bleeding, pneumonia, and intra-abdominal infection), cardiac surgery, cranial surgery, mandatory ventilation, prone position ventilation, fiberoptic bronchoscopy examination, tracheotomy, laboratory test at admission (D-dimer, prothrombin time, platelet count, antithrombin III, activated partial thromboplastin time), daily medication exposure and processes of care (sedative, acid inhibitors, blood transfusion, head-of-bed elevation, gastrointestinal decompression, rehabilitation exercise) and medications (sedatives, opioids, neuromuscular blockers, immunosuppressive agent, neuroleptic agents, antibiotics, expectorants, vasopressors, intestinal probiotics and neuroleptic agents), VAE, days from ICU admission to initiation of MV and duration of mechanical ventilation.

### Subgroup Analyses and Sensitivity Analyses

Among patients with D-dimer >5 mg/LFEU, regimens with antithrombotic (HR: 0.84, 95% CI: 0.72, 0.98) and anticoagulant (HR: 0.81, 95% CI: 0.68, 0.98) were associated with lower risk of VAEs than regimens without. However, among patients with D-dimer ≤5 mg/LFEU, the effect of antithrombotic, anticoagulant, and antiplatelet agent on VAEs was not statistically significant (Supplementary Table S1). With regarding to ICU mortality, regimens with antithrombosis agents and regimens with anticoagulant were associated with decreased risk of ICU mortality both among patients with D-dimer >5 mg/LFEU and ≤5 mg/LFEU (Supplementary Table S1).

Sensitivity analyses using alternative statistical models did not show change in interpretation. The result of complete cases analysis was consistent with primary analysis for the comparison of regimens with and without antithrombosis agents regarding hazards for VAEs, but with wider confidence intervals that crossed one (HR 0.88, 95% CI: 0.78, 1.01). The sensitivity analyses of ICU stays using alternative definition of comparison and alternative approach for missing data showed similar results, but the confidence interval less than one regarding the comparison of regimens with and without antiplatelet agents (Supplementary Table S2).

Additional analysis showed that antithrombosis agents were associated with decreased risk of VTE (HR 0.85, 95% CI:0.52, 1.39).

## Discussion

The results of this large observational study showed that pharmacological thromboprophylaxis was commonly administered in ICU patients on MV. Pharmacological thromboprophylaxis was associated with lower risk of VAEs and ICU mortality. The effects of different type of pharmacological thromboprophylaxis on clinical outcomes may differ: treatment of antithrombotic, anticoagulant, but not antiplatelet appear beneficial. Antithrombotic may be associated with decreased risk of VAEs among patients with D-dimer >5 mg/LFEU; however, the effect was not statistically significant among patients with D-dimer ≤5 mg/LFEU.

Similar to ours, several other studies have suggested that thrombosis prophylaxis may decrease the risk of adverse outcomes. A multicenter prospective observational study that included 630 patients receiving MV for at least 48 h showed that DVT prophylaxis was significantly associated with lower incidences of VAP (Croce et al., 2013). Another study on 175,665 ICU patients found that omission of early thromboprophylaxis may increase the risk of mortality for patients with critical illness (Ho et al., 2011). A possible reason may be that pharmacological thromboprophylaxis could decrease the risk of VTE, which may further result in adverse outcomes, such as respiratory deterioration, prolonged MV, and mortality. Two systematic review and meta-analyses showed significantly lower risk of DVT and PE in patients receiving thromboprophylaxis than those without (Alhazzani et al., 2013; Park et al., 2016). The in-hospital mortality was 50% higher among patients with DVT than those without (Jain and Schmidt, 1997; Ejaz et al., 2018), and PE was attributable to 4–11% cause of deaths (Laporte and Mismetti, 2010).

However, several other studies have not found a significant association between thromboprophylaxis and adverse outcomes. A case–control study including 110 VAE cases suggested that thrombosis prophylaxis did not reduce the risk of VAEs and IVACs (Lewis et al., 2014). Klompas et al. conducted a retrospective cohort study and showed that thromboembolism prophylaxis was not associated with decreased risk of VAEs, VAP, hospital stays, and mortality (Klompas et al., 2016). There are several potential reasons for these apparent inconsistencies in results. One reason may be due to the varied outcome definitions, especially for VAPs. For instance, Klompas et al. defined VAPs according to the new surveillance criteria proposed by US CDC, whereas Croce et al. used the traditional definition of VAP (Croce et al., 2013; Klompas et al., 2016). The correlation between VAE and VAP has been proved to be poor (Klompas, 2019). Secondly, the strategies for thromboembolism prophylaxis varied among studies and individual patients. Thromboprophylaxis included pharmacological and mechanical prophylaxis, and the effect of these two measures on preventing adverse events may differ (Park et al., 2016; Boddi and Peris, 2017). A meta-analysis including 12 trials suggested that pharmacological prophylaxis with UFH and LMWH significantly decreased the incidence of DVT. Mechanical thromboprophylaxis, however, was not associated with a significant reduction of DVT risk (Park et al., 2016). Although most previous studies involved both pharmacological and mechanical prophylaxis, the detailed measures and compliance with thromboprophylaxis among studies likely varied (Manoucheri and Fallahi, 2015; Ejaz et al., 2018). In addition, these studies involved patients with different clinical features, such as coagulation function. The effect of thromboprophylaxis on clinical outcomes may differ among patients with different coagulation function (Goodwin and Hoffman, 2011). Patients with hypercoagulability may more likely to benefit from thromboprophylaxis. Our study also showed that antithrombotic was associated with lower risk VAEs among patients with elevated D-dimer.

### Strengths and Limitations

This study has several strengths. First, our study was conducted based on a multisource database, which contained complete and accurate information regarding HAI in the ICU setting. We included a relatively large number of patients in addition to identifying a large number of VAEs. Validation of outcomes showed a high level of accuracy. Second, we considered time-varying terms for daily pharmacological thromboprophylaxis exposures and used competing risk models to handling the competing risks for ICU discharge alive versus ICU mortality.

Our study also has some limitations. First, the results should be interpreted with caution given the nature of the retrospective observational study. Although we adjusted an extensive array of factors, unmeasured residual confounding factors may still be present. Second, data for some variables were missing. Third, this study was conducted using data from a homogeneous healthcare system, and the findings of our study may not be generalizable to other healthcare settings. Fourthly, only few patients developed VTE, and the inference on the effect of VTE is weakened.

## Conclusion

Antithrombosis agents were associated with a lower risk of VAEs and ICU mortality than regimens without antithrombosis agents. Anticoagulation but not antiplatelet agents appeared beneficial. The effects appeared comparable on comparing UFH vs. LMWH. Large, rigorous, randomized trials are needed to validate these results.

## Data Availability

The original contributions presented in the study are included in the article/Supplementary Material, and further inquiries can be directed to the corresponding authors.

## Abbreviations

APACHE II, Acute physiology and chronic health evaluation; CDC, Centers for Disease Control and Prevention; CI, Confidence interval; DVT, Deep vein thrombosis; EMR, Electronic medical record; FiO_2_, Fraction of inspired oxygen; HAI, Healthcare-associated infections; HR, Hazard ratio; ICU, Intensive care units; IVAC, Infection-related ventilator-associated complication; LMWH, Low molecular weight heparin; MV, Mechanical ventilation; PE, Pulmonary embolism; PEEP, Positive end-expiratory pressure; PVAP, Possible ventilator-associated pneumonia; UFH, Unfractionated heparin; VAC, Ventilator-associated condition; VAEs, Ventilator-associated events; VAP, Ventilator-associated pneumonia; VTE, Venous thromboembolism; WCH, West China Hospital.
