# Supplementary material for: Associations Between Antithrombosis and Ventilator-Associated Events, ICU Stays, and Mortality Among Mechanically Ventilated Patients: A Registry-Based Cohort Study
Source: Front Pharmacol. 2022 Jul 18;13:891178. doi: 10.3389/fphar.2022.891178 (PMC9339989; doi:10.3389/fphar.2022.891178)
Supplement: Supplementary file 1 [file DataSheet1.docx]

**Supplementary** **Table 1. Association between antithrombotic agents and VTE**

|  | **Crude model for VTE** | | **Adjusted model for VTE** | |
| --- | --- | --- | --- | --- |
|  | HR (95% CI) | P value | HR (95% CI) | P value |
| Antithrombotic agents vs regimens without antithrombotic agents | 0.68 (0.44, 1.05) | 0.087 | 0.85 (0.52, 1.39) | 0.526 |
| Anticoagulant agents vs regimens without antithrombotic agents | 1.12 (0.54, 2.33) | 0.760 | 1.14 (0.52, 2.48) | 0.742 |
| Antiplatelet agents vs regimens without antithrombotic agents | 0.61 (0.37, 1.02) | 0.061 | 0.79 (0.43, 1.42) | 0.431 |

ICU: intensive care units; HR: hazard ratio; VTE: venous thromboembolism.

Model adjusted for: age, sex, acute physiology and chronic health evaluation (APACHE) Ⅱ score, ICU type (general, surgical, neurological, respiratory, thoracic surgery and pediatric ICU), comorbidities or condition (diabetes, hypertension, heart failure, kidney failure, liver failure, ischemic heart disease, cerebrovascular diseases, chronic obstructive pulmonary disease, pulmonary vascular diseases, malignant tumor, trauma, acute respiratory distress syndrome, shock, gastrointestinal bleeding, pneumonia, and intra-abdominal infection), cardiac surgery, cranial surgery, mandatory ventilation, prone position ventilation, fiberoptic bronchoscopy examination, tracheotomy, laboratory test at admission (D-dimer, prothrombin time, platelet count, antithrombin III, activated partial thromboplastin time), daily medication exposure and processes of care (sedative, acid inhibitors, blood transfusion, head-of-bed elevation, gastrointestinal decompression, rehabilitation exercise) and medications (sedatives, opioids, neuromuscular blockers, immunosuppressive agent, neuroleptic agents, antibiotics, expectorants, vasopressors, intestinal probiotics and neuroleptic agents), VAE, days from ICU admission to initiation of MV and duration of mechanical ventilation.

**Supplementary** **Table 2. Subgroup analyses of ICU mortality and ICU stays by D-dimer**

| **Subgroup analysis** | **Adjusted HR (95% CI)** | **P value** |
| --- | --- | --- |
| **Ventilator-associated events** |  |  |
| **D-dimer** ≤ **5 mg/L** |  |  |
| Antithrombotic agents vs regimens without antithrombotic agents | 0.92 (0.78, 1.10) | 0.377 |
| Anticoagulant agents vs regimens without antithrombotic agents | 0.92 (0.76, 1.13) | 0.454 |
| Antiplatelet agents vs regimens without antithrombotic agents | 0.94 (0.71, 1.23) | 0.645 |
| **D-dimer > 5 mg/L** |  |  |
| Antithrombotic agents vs regimens without antithrombotic agents | 0.84 (0.72, 0.98) | 0.032 |
| Anticoagulant agents vs regimens without antithrombotic agents | 0.81 (0.68, 0.98) | 0.027 |
| Antiplatelet agents vs regimens without antithrombotic agents | 0.90 (0.69, 1.17) | 0.418 |
| **ICU mortality** |  |  |
| **D-dimer** ≤ **5 mg/L** |  |  |
| Antithrombotic agents vs regimens without antithrombotic agents | 0.65 (0.49, 0.86) | 0.002 |
| Anticoagulant agents vs regimens without antithrombotic agents | 0.54 (0.39, 0.75) | <0.001 |
| Antiplatelet agents vs regimens without antithrombotic agents | 0.81 (0.51, 1.26) | 0.348 |
| **D-dimer > 5 mg/L** |  |  |
| Antithrombotic agents vs regimens without antithrombotic agents | 0.76 (0.60, 0.96) | 0.02 |
| Anticoagulant agents vs regimens without antithrombotic agents | 0.69 (0.53, 0.90) | 0.005 |
| Antiplatelet agents vs regimens without antithrombotic agents | 1.29 (0.89, 1.86) | 0.174 |
| **ICU stays** |  |  |
| **D-dimer** ≤ **5 mg/L** |  |  |
| Antithrombotic agents vs regimens without antithrombotic agents | 0.96 (0.84, 1.11) | 0.608 |
| Anticoagulant agents vs regimens without antithrombotic agents | 1.05 (0.90, 1.22) | 0.516 |
| Antiplatelet agents vs regimens without antithrombotic agents | 0.77 (0.62, 0.96) | 0.022 |
| **D-dimer > 5 mg/L** |  |  |
| Antithrombotic agents vs regimens without antithrombotic agents | 1.077 (0.95, 1.22) | 0.261 |
| Anticoagulant agents vs regimens without antithrombotic agents | 1.14 (1.00, 1.31) | 0.054 |
| Antiplatelet agents vs regimens without antithrombotic agents | 0.78 (0.63, 0.97) | 0.026 |

ICU: intensive care units; HR: hazard ratio.

Model adjusted for: age, sex, acute physiology and chronic health evaluation (APACHE) Ⅱ score, ICU type (general, surgical, neurological, respiratory, thoracic surgery and pediatric ICU), comorbidities or condition (diabetes, hypertension, heart failure, kidney failure, liver failure, ischemic heart disease, cerebrovascular diseases, chronic obstructive pulmonary disease, pulmonary vascular diseases, malignant tumor, trauma, acute respiratory distress syndrome, shock, gastrointestinal bleeding, pneumonia, and intra-abdominal infection), cardiac surgery, cranial surgery, mandatory ventilation, prone position ventilation, fiberoptic bronchoscopy examination, tracheotomy, laboratory test at admission (D-dimer, prothrombin time, platelet count, antithrombin III, activated partial thromboplastin time), daily medication exposure and processes of care (sedative, acid inhibitors, blood transfusion, head-of-bed elevation, gastrointestinal decompression, rehabilitation exercise) and medications (sedatives, opioids, neuromuscular blockers, immunosuppressive agent, neuroleptic agents, antibiotics, expectorants, vasopressors, intestinal probiotics and neuroleptic agents), VAE, days from ICU admission to initiation of MV and duration of mechanical ventilation.

**Supplementary** **Table 3. Sensitivity analyses** **using alternative definition and statistical model for hazard ratios regarding ventilator-associated events, ICU mortality and ICU stay**

|  | **Adjusted model for VAEs** | | **Adjusted model for ICU mortality** | | **Adjusted model for ICU stays** | |
| --- | --- | --- | --- | --- | --- | --- |
|  | **HR (95% CI)** | **P value** | **HR (95% CI)** | **P value** | **HR (95% CI)** | **P value** |
| **Alternative statistical models** |  |  |  |  |  |  |
| Antithrombotic agents vs regimens without antithrombotic agents | 0.86 (0.77, 0.97) | 0.013 | 0.71 (0.60, 0.84) | <0.001 | 1.08 (0.98, 1.18) | 0.12 |
| Anticoagulant agents vs regimens without antithrombotic agents | 0.85 (0.74, 0.96) | 0.013 | 0.61 (0.50, 0.74) | <0.001 | 1.15 (1.05, 1.27) | 0.004 |
| Antiplatelet agents vs regimens without antithrombotic agents | 0.91 (0.75, 1.09) | 0.314 | 1.10 (0.85, 1.43) | 0.48 | 0.88 (0.77, 1.02) | 0.082 |
| Anticoagulant agents vs antiplatelet agents | 0.92 (0.72, 1.17) | 0.5 | 0.52 (0.37, 0.73) | <0.001 | 1.44 (1.18, 1.74) | <0.001 |
| LMWH vs UFH | 1.09 (0.85, 1.39) | 0.491 | 0.92 (0.62, 1.37) | 0.679 | 1.01 (0.83, 1.22) | 0.912 |
| **Complete cases analyses** |  |  |  |  |  |  |
| Antithrombotic agents vs regimens without antithrombotic agents | 0.88 (0.78, 1.01) | 0.064 | 0.72 (0.60, 0.87) | 0.001 | 1.03 (0.93, 1.14) | 0.532 |
| Anticoagulant agents vs regimens without antithrombotic agents | 0.86 (0.74, 0.99) | 0.042 | 0.63 (0.51, 0.78) | <0.001 | 1.11 (1.00, 1.24) | 0.051 |
| Antiplatelet agents vs regimens without antithrombotic agents | 0.90 (0.73, 1.12) | 0.363 | 1.21 (0.91, 1.61) | 0.187 | 0.76 (0.64, 0.91) | 0.002 |
| Anticoagulant agents vs antiplatelet agents | 0.91 (0.69, 1.20) | 0.499 | 0.49 (0.34, 0.72) | <0.001 | 1.55 (1.23, 1.96) | <0.001 |
| LMWH vs UFH | 1.08 (0.81, 1.44) | 0.6 | 0.84 (0.53, 1.30) | 0.428 | 1.07 (0.80, 1.42) | 0.663 |
| **Alternative definition of comparison** |  |  |  |  |  |  |
| Anticoagulant agents vs regimens without anticoagulant agents | 0.86 (0.76, 0.98) | 0.023 | 0.62 (0.51, 0.76) | <0.001 | 1.17 (1.06, 1.29) | 0.001 |
| Antiplatelet agents vs regimens without antiplatelet agents | 0.95 (0.79, 1.15) | 0.609 | 1.24 (0.95, 1.60) | 0.113 | 0.83 (0.73, 0.95) | 0.007 |

VAE: ventilator-associated events; ICU: intensive care units; HR: hazard ratio; UFH: unfractionated heparin; LWMH: low molecular weight heparin.

For complete cases analyses and alternative definition of comparison, model adjusted for: age, sex, acute physiology and chronic health evaluation (APACHE) Ⅱ score; ICU type (general, surgical, neurological, respiratory, thoracic surgery and pediatric ICU), comorbidities or condition (diabetes, hypertension, heart failure, kidney failure, liver failure, ischemic heart disease, cerebrovascular diseases, chronic obstructive pulmonary disease, pulmonary vascular diseases, malignant tumor, trauma, acute respiratory distress syndrome, shock, gastrointestinal bleeding, pneumonia, and intra-abdominal infection), cardiac surgery, cranial surgery, mandatory ventilation, prone position ventilation, fiberoptic bronchoscopy examination, tracheotomy, laboratory test at admission (D-dimer, prothrombin time, platelet count, antithrombin III, activated partial thromboplastin time), daily medication exposure and processes of care (sedative, acid inhibitors, blood transfusion, head-of-bed elevation, gastrointestinal decompression, rehabilitation exercise) and medications (sedatives, opioids, neuromuscular blockers, immunosuppressive agent, neuroleptic agents, antibiotics, expectorants, vasopressors, intestinal probiotics and neuroleptic agents), VAE, days from ICU admission to initiation of MV and duration of mechanical ventilation. For alternative statistical model, model without adjusting prothrombin time, platelet count, antithrombin III, activated partial thromboplastin time
